# Additional chemotherapy improved local control and overall survival after stereotactic body radiation therapy for patients with oligo-recurrence

**DOI:** 10.1186/s13014-018-1031-0

**Published:** 2018-04-23

**Authors:** Masaki Nakamura, Naoki Hashimoto, Hiroshi Mayahara, Haruka Uezono, Aya Harada, Ryo Nishikawa, Yoshiro Matsuo, Hiroki Kawaguchi, Hideki Nishimura

**Affiliations:** 1 Department of Radiation Oncology, Kobe Minimally invasive Cancer Center, 8-5-1, Minatojima Nakamachi, Chuo-ku, Kobe, Hyogo 650-0046 Japan; 2 Division of Radiation Oncology and Particle Therapy, National Cancer Center Hospital East, 6-5-1, Kashiwanoha, Kashiwa, Chiba, 277-8577 Japan; 3 Department of Radiation Oncology, University of Florida Proton Therapy Institute, 2015 N Jefferson St, Jacksonville, FL 32206 USA; 4 Division of Radiation Oncology, Kobe University Graduate School of Medicine, 7-5-2 Kusunoki-Cho, Chuo-ku, Kobe, Hyogo 650-0017 Japan; 5 Department of Radiology, Hyogo Ion Beam Medical Center, 1-2-1, Koto, Shingu-cho, Tatsuno, Hyogo 679-5165 Japan

**Keywords:** Oligo, Meta, Recurrence, SBRT, Lung, Liver

## Abstract

**Background:**

Oligo-recurrence has been considered to confer improved prognosis than other oligometastatic conditions, and stereotactic body radiation therapy (SBRT) is considered as an option of local therapy for lung or liver metastases. The purpose of this study was to investigate the efficacy and safety of SBRT for lung and liver oligo-recurrent lesions and evaluate predictive factors for local control and prognosis.

**Methods:**

This retrospective study included patients who presented with 1–3 matachronous lung or liver metastases, and treated with SBRT between May 2013 and March 2016 at a single institution. All patients harbored a controlled primary lesion. Patients with < 6 months of follow-up were excluded. Local control, progression free survival, and overall survival rates were analyzed according to the Kaplan–Meier product limit method. Univariable log-rank and multivariable Cox regression analyses were performed to clarify predictive factors for local control and prognosis. Toxicity was graded according to the Common Terminology Criteria for Adverse Events, version 4.0.

**Results:**

Seventy-six patients with a total of 70 and 44 lung and liver lesions were included. The median follow-up period was 21 (range, 7–43) months. The 1-year local control, progression-free survival and overall survival rates were 89, 38 and 96%, respectively. Smaller gross tumor volume and additional chemotherapy after SBRT were significant predictive factors for better local control (*p* = 0.005 and *p* = 0.047), and the presence of a single metastatic lesion was a significant factor of good progression free survival (*p* = 0.008). Additional chemotherapy after SBRT was not a significant predictive factor but conferred to better overall survival (*p* = 0.078). Among colorectal cancer patients, post SBRT chemotherapy was significantly associated with better OS (*p* = 0.025). Over grade 3 adverse event was seen in only one patient.

**Conclusion:**

SBRT is a safe and effective treatment for patients with lung and liver oligo-recurrence. Additional chemotherapy after SBRT improved local control, and single metastatic lesion was a significant predictive factor of better PFS in this study. Among colorectal cancer patients, additional chemotherapy after SBRT significantly associated better OS.

## Background

The condition of distant metastases from cancer has generally been regarded as a systemic disease [1], and the role of local therapy remains questionable. The condition of oligometastases, wherein the number and sites of metastatic tumors are limited [2], has been recently highlighted in the treatment of metastatic tumors. Metastatic disease is a leading cause of cancer mortality, but localized forms of cancer treatment may be effective in patients with oligometastases [3].

Oligo-recurrence is defined as the state in which patients with cancer have ≤5 metastatic or recurrent lesions with controlled primary lesion, and has been considered to confer improved prognosis than sync-oligometastases have ≤5 metastatic or recurrent lesions with active primary lesions [4]. However, information on the specific oligometastatic cases in which local treatment may be effective remains insufficient and the selection of adaptation cases for local treatment is of particular concern to oncologists.

The lungs and liver are common sites for metastatic seeding, and surgery is a standard treatment option, with good results in terms of local control and survival [5, 6]. Stereotactic body radiation therapy (SBRT) is a method for delivering high doses of radiation to the target, utilizing a small number of fractions with a high degree of precision within the body. It is an option for local therapy, and phase II studies utilizing this therapy for lung or liver metastases have reported good local control (LC) [7, 8].

In this study, we aimed to investigate the efficacy and safety of SBRT for lung and liver oligo-recurrent lesions and evaluate predictive factors for LC and prognosis.

## Methods

This study included patients who were treated with SBRT between May 2013 and March 2016 at the Kobe Minimally Invasive Cancer Center. The study cohort included patients who presented with 1–3 metachronous lung or liver metastases with a controlled primary lesion. Patients with < 6 months of follow-up were excluded. Pretreatment diagnosis was performed using computed tomography (CT), magnetic resonance (MR) imaging, or ^18^F-fluorodeoxyglucose positron emission tomography scans (FDG-PET). Histological proof of metastatic lesions was not indispensable. All study participants provided informed, written consent. The study protocol was approved by the Research Ethics Committee of our institution (reference number: 2018-[kenkyu01]-09). The research was conducted in accordance with the 1964 Declaration of Helsinki and its later amendments.

All SBRT procedures were performed with the CyberKnife® VSI™ System (Accuracy Inc., Sunnyvale, CA, USA). The CyberKnife SBRT method for lung tumors was performed as previously described [9]. A spine tracking system was used during the treatment of tumors that were located in the apical region and exhibited small respiratory movement. The spine tracking system is able to detect and track the bony anatomy of the spine to guide beam targeting without synchronizing respiratory movement. A directed tumor tracking system was used during the treatment of tumors that were over 15 mm in diameter, located in the visible in the orthogonal X-ray images created by the CyberKnife VSI System. Other tumors were treated with fiducial tracking system. In this system, the intravascular or transcutaneous method was used to place one fiducial marker close to the tumor. The motion of red light-emitting diodes attached to the patient chest wall was then registered and correlated to the location of the implanted fiducial, as determined by a series of orthogonal X-ray images taken during respiration.

The patients were immobilized with a Vac-Lok cushion (CIVCO, USA). A thin-sliced, four-dimensional CT scan was acquired with 1-mm slices. Contrast enhancement was performed as required. In the treatment of liver lesions, contrast-enhanced MR images were indispensable unless contraindicated. The organs at risk (i.e., the spinal cord, normal lung or liver tissue, heart, duodenum, stomach, kidney, and esophagus) were contoured on the CT scan at the resting respiratory level. Gross tumor volumes (GTVs) were contoured on each phase of the four-dimensional CT scans registered with the fiducial marker in the fiducial tracking system, the tumor in the tumor tracking system, and the vertebral body in the spine tracking system. The internal target volume was defined as a fusion of all GTVs at each phase of the four-dimensional CT scans. For lung tumors, the planning target volume (PTV) equaled the internal target volume plus 2–6 mm; this heterogeneous margin was based on tracking methods. For liver tumors, PTV equaled the internal target volume plus 4 mm; all liver tumors were treated with fiducial tracking methods. The MultiPlan 4.6.0 treatment planning software (Accuracy Inc., Sunnydale, CA, USA) was used to plan the treatments. Radiation doses were calculated using the Monte Carlo algorithm in lung tumors and the ray tracing algorithm in liver tumors. Treatment consisted of a 6-MV radiation beam using one or two circular collimator cones. The prescribed radiation dose delivered to the PTV was prescribed to the 75–85% isodose line, covering ≥95% of the PTV. The standard prescription dose was 60Gy at our institute. However, in order to respect the constraints of the organs at risk, an adjustment of prescription dose was permitted. For patients with lung lesions, 3 or 4 fractionated therapy was performed. If PTV overlapped the hilar pulmonary vein or main bronchus, 8 fractionated therapy was selected. For patients with liver lesions, 4 fractionated therapy was performed. If PTV overlapped the right or left portal vein, 8 fractionated therapy was selected.

Patients initially underwent a follow-up CT 2–3 months following the SBRT. All patients underwent serial CT at 2–3-month intervals. Additional imaging, such as MR imaging or FDG-PET, was also performed if clinically indicated. All patients were registered until death or loss to follow-up. Local recurrence of lesions was defined as the evidence of tumor volume enlargement or the appearance of a new lesion in the PTV. Progression-free survival (PFS) was defined as the absence of local, regional, or distant progression. Suspected recurrence or new lesions were confirmed by at least two imaging modalities.

Local control, PFS, and overall survival (OS) rates were analyzed according to the Kaplan–Meier product limit method with 95% confidence intervals (CIs) and were measured from the date of radiotherapy initiation. Univariable log-rank and multivariable Cox regression analyses were performed. Cut-off values of continuous valuables were calculated by receiver-operating characteristic curve (ROC) analyses. The Cox regression analysis simultaneously included factors that had shown associations (*p* < 0.100) in the log-rank analyses. When faced with factors that were correlated with each other, we selected the factor with the highest area under the curve in ROC analyses. All statistical analyses were conducted using R software, version 3.4.0 (The R Foundation, Vienna, Austria). All tests were two-sided and *p*-values of < 0.05 were considered statistically significant. Toxicity was graded according to the Common Terminology Criteria for Adverse Events, version 4.0.

## Results

**Fig. 1:**
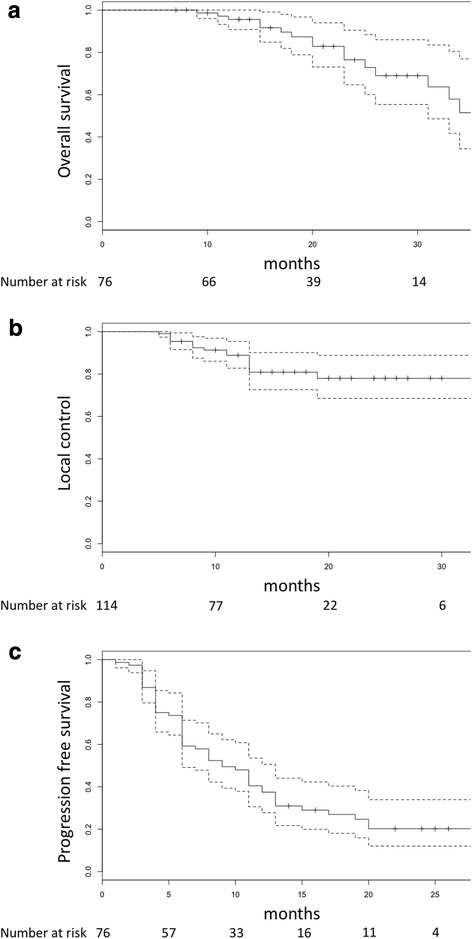
Kaplan-Meier curves of (**a**) overall survival, (**b**) local control, (**c**) progression free survival

Eighty-five patients who were treated with SBRT for oligo-recurrent lung and liver lesions between May 2013 and March 2016 at our institute were included. Nine patients were excluded because of loss to follow-up within 6 months. In total, 76 patients with a total of 70 lung lesions and 44 liver lesions were included. There was no patient whose metastatic lesions left untreated. Patient and lesion characteristics are provided in Table 1. Primary tumors in the patients were observed in the colorectum (*n* = 46 patients; 61%), gastric region (*n* = 5 patients; 7%), ovaries (*n* = 2 patients; 2.5%), esophagus (*n* = 2), pancreas (*n* = 2), lung (*n* = 2), liver (*n* = 2), duodenum (*n* = 2), breast (*n* = 2), kidney (*n* = 2), and other regions (*n* = 9 patients; 12%). The total radiation dose range was 48–64 Gy in 3, 4, or 8 equal fractions. Nine lesions (7.9%) were treated with 3 fractions, 80 lesions (70.2%) with 4 fractions, and 25 lesions (21.9%) with 8 fractions. The median follow-up period was 21 months (range, 7–43) and 16 patients died during follow-up. Death was related to development of cancer in 11 patients and other disease in 1 patient. The cause of death was unknown in the remaining 4 patients. The 1- and 2-year OS rates were 96% (95% CI: 91–100) and 76% (95% CI: 64–90), respectively (Fig. 1a).

**Table 1:** Patient and lesion characteristics

	Patient total (*n* = 76)	Lesion total (*n* = 114)
Age, Median (Range), years	69 (37–91)	
Gender, n (%)		
ᅟMale	44 (58%)	
ᅟFemale	32 (42%)	
ECOG PS, n (%)		
ᅟ0	42 (55%)	
ᅟ1–2	34 (45%)	
Single lesion, n (%)	57 (75%)	
Histology of primary lesion, n (%)		
ᅟCRC	46 (61%)	73 (64%)
ᅟNon CRC	30 (39%)	41 (36%)
Previous local therapy, n (%)	8 (11%)	
Previous chemotherapy, n (%)	52 (68%)	83 (73%)
Posterior chemotherapy, n (%)	19 (25%)	30 (26%)
Metastatic location, n (%)		
ᅟLung		70 (61%)
ᅟLiver		44 (39%)
MTD, Median (Range), mm		19 (5–57)
GTV, Median (Range), ml		3.2 (0.2–106.8)
PTV dose BED_10_, Median (Range), Gy		150 (81.3–180)

*ECOG* Eastern Cooperative Oncology Group, *PS* performance status, *CRC* colorectal cancer, *MTD* maximum tumor diameter, *GTV* gross tumor volume, *PTV* planning target volume, *BED* biological effective dose

Seven of the 70 lung lesions and 10 of the 44 liver lesions experienced local failure. The 1- and 2-year LC rates were 89% (95% CI: 83–95) and 78% (95% CI: 69–89), respectively (Fig. 1b). Predictive factors for LC are provided in Table 2. Cut-off value of GTV was defined as 7 ml by ROC analysis. Smaller GTV and addition of post SBRT chemotherapy were significant predictive factors for better local control (*p* = 0.005, *p* = 0.047). New metastatic lesions appeared in 51 patients. The 1- and 2-year PFS rates were 38% (95% CI: 28–51) and 20% (95% CI: 12–34), respectively (Fig. 1c). Predictive factors for PFS and OS are provided in Table 3. The presence of a single metastatic lesion was a significant factor of improved PFS (*p* = 0.010) and addition of post SBRT chemotherapy conferred to better OS (*p* = 0.078). Of the 19 patients received post SBRT chemotherapy, 18 were colorectal cancer patients. So, additional analyses were carried out in 46 colorectal cancer patients. The results of univariate and multivariate analyses shown only post SBRT chemotherapy was significantly associated with better OS among colorectal cancer patients (*p* = 0.007 and *p* = 0.025, respectively).

**Table 2:** The univariate and multivariate analysis of factors related to local control

	Local control		
	Univariate (*p* value)	Multivariate (*p* value)	Hazard ratio (95%CI)
Histology: CRC vs others	0.556		
Previous chemotherapy: yes vs no	0.406		
Posterior chemotherapy: yes vs no	0.020	0.047	0.13 (0.02–0.97)
Location: lung vs liver	0.044	0.569	0.74 (0.25–2.13)
MTD: ≥ 21 vs < 21	0.010		
GTV: ≥ 7 vs < 7	0.002	0.005	4.62 (1.57–13.58)
PTV BED_10_ dose: 95 ≥ vs < 95	0.582		

*CRC* colorectal cancer, *MTD* maximum tumor diameter, *GTV* gross tumor volume, *PTV* planning target volume, *BED* biological effective dose, *CI* confidence interval

**Table 3:** The univariate and multivariate analysis of factors related to progression free survival and overall survival

	Progression free survival			Overall survival		
	Univariate (*p* value)	Multivariate (*p* value)	Hazard ratio (95%CI)	Univariate (*p* value)	Multivariate (*p* value)	Hazard ratio (95%CI)
Age: ≥ 70 vs < 70	0.195			0.638		
Gender: male vs female	0.607			0.774		
ECOG PS: 0 vs 1–2	0.435			0.866		
Lesion number: single vs multiple	0.008	0.010	0.46 (0.26–0.83)	0.053	0.112	0.43 (0.15–1.22)
Histology: CRC vs others	0.496			0.935		
Previous chemotherapy: yes vs no	0.378			0.927		
Posterior chemotherapy: yes vs no	0.088	0.126	1.60 (0.88–2.93)	0.035	0.078	2.72 (0.90–8.27)

*ECOG* Eastern Cooperative Oncology Group, *PS* performance status, *CRC* colorectal cancer, *CI* confidence interval

Grade 3 radiation dermatitis was observed in 1 patient. This patient underwent two courses of SBRT for liver lesions and dermatitis caused by overlap of two radiation fields. Overlapped skin dose was total 56 Gy in 4 fractions. Of the 28 patients who underwent more than 2 courses of SBRT, over grade 2 adverse event due to radiation dose overlap was observed only this case. Grade 2 adverse events were observed in 2 patients. One developed chest wall pain. This patient underwent 2 courses of SBRT for lung lesions, but there was no overlap of dose distribution in the chest wall. Another developed radiation pneumonitis. This patient underwent only 1 course of SBRT. Grade 1 adverse events were observed in 53 patients. Regarding toxicities that were related to the method of fiducial marker placement, one grade 1 intra-pelvic hematoma and one stomachache were observed.

## Discussion

We reported a single-institutional retrospective study that investigated and analyzed patients with oligo-recurrent lesions in the lungs and liver treated with SBRT using a robotic radiosurgery system. For the 114 lesions of 76 patients treated, the 1- year LC, PFS and OS rates were 89, 38 and 96%, respectively. Grade 3 and higher-grade toxicity occurred in only 1% (2/76) of the patients. These results were almost identical to the results reported in other literature (Table 4).

**Table 4:** Recent reports on stereotactic body radiation therapy for lung or liver metastases

Aurhor (reference)	Lesion /Patients, n	CRC rate, %	Median f/u period, m	Median MTD, mm	Median GTV, mL	LC, %		PFS, %		OS, %		AE ≥ G3, %
						1 yr	2 yr	1 yr	2 yr	1 yr	2 yr	
Rusthoven (7)	63/ 38	24	15	NA	4.2	100	96	NA	NA	NA	39	8
Rurthoven (8)	63/ 47	32	16	27	14.9	95	92	NA	NA	NA	30	2
Binkley (10)	122/ 77	21	22	NA	3.7	91	84	NA	25	94	75	0
Andratschke (13)	91/ 52	42	17	28	NA	NA	82	35	18	70	45	1.9(≥G2)
Ricard (14)	77/ 61	21	20	NA	NA	NA	89	NA	32	NA	67	2
Filippi (15)	59/ 40	100	46	15	NA	93	NA	49	27	84	73	7.5(≥G2)
Agolli (16)	69/ 44	100	36	14	NA	NA	NA	NA	20	NA	68	0
Katz (17)	74/ 69	29	15	27	NA	76	57	24	NA	78	37	0
Scorsetti (18)	52/ 42	100	24	35	NA	95	91	NA	46	NA	65	0
Joo (19)	103/ 70	100	34	29	NA	93	89	NA	35	NA	75	NA
Wang (25)	134/ 95	18	17	NA	14.6	98	91	51	29	83	61	3
Vautrauers-Dewas (26)	62/ 42	67	14	25	13	90	86	NA	NA	94	48	6.3
Present study	114/ 76	61	21	19	3.2	89	78	38	20	96	76	1.3

*CRC* colorectal cancer, *MTD* maximum tumor diameter, *GTV* gross tumor volume, *LC* local control, *PFS* progression free survival, *OS* overall survival, *AE* adverse event, NA:

Our results indicated that the LC rate of large GTV (≥ 7 ml) lesions were significantly lower than those of small GTV (< 7 ml) lesions (*p* = 0.005). The relationship between tumor volume and LC has been reported previously [10, 11]. Other reports described primary histology and dosimetric factors as being related to LC. Ahmed et al. described the 1- and 2-year LC rates for colorectal lesions were 79 and 59%, compared with 100% for non-colorectal lesions [12]. Andratschke et al. described the 2-year LC rates for PTV prescription BED10 > 86.1 Gy was 96.6% and for ≤86.1 Gy10 68.1%, the difference being significant [13]. In our study, significant relationship was not observed between histology of CRC and LC. Furthermore, it is difficult to accurately evaluate the exact dosimetric levels because different dose calculation algorithms are used for the lungs and liver treatment.

In this study, PFS was shown to be poor compared to superior LC and OS. This tendency was recognized in other reports as well. The 1- and 2-year PFS rates were 24–51 and 18–46% in other reports, respectively [13–20]. Only 12% (9/76) of the patients did not exhibit disease progression or death 20 months after SBRT in this study; these patients all harbored a single metastatic lesion at the time of SBRT consultation. The presence of a single metastatic lesion was a significant predictive factor of improved PFS. However, many of the patients with a single metastatic lesion (48/57) experienced disease progression. This may be due to the fact that patients with the state of systemic disease were included in the treatment group. Furthermore, a Japanese prospective study of resection for oligometastases of non-small cell lung cancer included 18% (6/34) non-malignant lesions [21]. There may be limits to imaging-guided evaluation for oligometastatic conditions. Recently, studies have indicated that biomarkers are useful for the evaluation of tumor burden [22, 23]. The determination of molecular markers to distinguish oligometastatic from polymetastatic diseases are ongoing [24]. Wong et al. described a candidate classifier using expression levels of three microRNAs (miR-23b, miR-449a, and miR-449b) to predict survival after oligometastasis-directed SBRT [25]. Although still in the research stage, evaluation of oligometastatic conditions using liquid biopsy is expected.

There was a report that patients who received chemotherapy before SBRT had better local control [26]. On the other hand, other reports described contrary results [27, 28]. Klement et al. explained prior chemotherapy affect determination of target volume or reduction of radiation sensitivity [27]. In our study, prior chemotherapy didn’t contribute tumor control, but additional chemotherapy after SBRT related to better LC and OS. Particularly, additional chemotherapy after SBRT improved OS among colorectal cancer patients. The strategy of combining surgery with chemotherapy is adopted for liver metastases in colorectal cancer [29]. This strategy may also be applicable to radiotherapy. However, patients who received chemotherapy for metastatic condition had heterogeneity regarding chemotherapy regimen or number of chemotherapy courses. Further analysis is needed to clarify efficacy of combination therapy of chemotherapy and SBRT using a larger number of more homogeneous patients.

Limitations of this study include its retrospective nature and relatively limited follow-up time. Longer follow-up periods are needed for slow-growing tumor subtypes. Additionally, this study was limited by the heterogeneity of tumor histology. The combined analysis of different types of tumor histology with various prior treatments makes thorough evaluation of treatment efficacy difficult.

## Conclusions

SBRT is a safe and effective treatment for patients with lung and liver oligo-recurrence. Additional chemotherapy after SBRT improved local control, and single metastatic lesion was a significant predictive factor of better PFS in this study. Among colorectal cancer patients, additional chemotherapy after SBRT significantly associated better OS.

## Abbreviations

CIs: Confidence intervals
CT: Computed tomography
FDG-PET: ^18^F-fluorodeoxyglucose positron emission tomography scans
GTV: Gross tumor volume
LC: Local control
MR: Magnetic resonance
OS: Overall survival
PFS: Progression free survival
PTV: Planning target volume
SBRT: Stereotactic body radiation therapy
